# Emotion Recognition Model of EEG Signals Based on Double Attention Mechanism

**DOI:** 10.3390/brainsci14121289

**Published:** 2024-12-21

**Authors:** Yahong Ma, Zhentao Huang, Yuyao Yang, Shanwen Zhang, Qi Dong, Rongrong Wang, Liangliang Hu

**Affiliations:** 1 Xi’an Key Laboratory of High Pricision Industrial Intelligent Vision Measurement Technology, School of Electronic Information, Xijing University, Xi’an 710123, China; yyy11171999@outlook.com (Y.Y.); wjdw716@163.com (S.Z.); wangrongrong@xijing.edu.cn (R.W.); 2 Affiliation College of Computer Science and Technology, Chongqing University of Posts and Telecommunications, Chongqing 400065, China; 3 School of Mathematics and Statistics, Zhengzhou University, Zhengzhou 710003, China; qid004841@gmail.com; 4 West China Institute of Children’s Brain and Cognition, Chongqing University of Education, Chongqing 400065, China; hull@cque.edu.cn

**Keywords:** brain-computer interface, brainwave signals, emotion recognition, convolutional neural network, bidirectional long short-term memory network

## Abstract

Background: Emotions play a crucial role in people’s lives, profoundly affecting their cognition, decision-making, and interpersonal communication. Emotion recognition based on brain signals has become a significant challenge in the fields of affective computing and human-computer interaction. Methods: Addressing the issue of inaccurate feature extraction and low accuracy of existing deep learning models in emotion recognition, this paper proposes a multi-channel automatic classification model for emotion EEG signals named DACB, which is based on dual attention mechanisms, convolutional neural networks, and bidirectional long short-term memory networks. DACB extracts features in both temporal and spatial dimensions, incorporating not only convolutional neural networks but also SE attention mechanism modules for learning the importance of different channel features, thereby enhancing the network’s performance. DACB also introduces dot product attention mechanisms to learn the importance of spatial and temporal features, effectively improving the model’s accuracy. Results: The accuracy of this method in single-shot validation tests on the SEED-IV and DREAMER (Valence-Arousal-Dominance three-classification) datasets is 99.96% and 87.52%, 90.06%, and 89.05%, respectively. In 10-fold cross-validation tests, the accuracy is 99.73% and 84.26%, 85.40%, and 85.02%, outperforming other models. Conclusions: This demonstrates that the DACB model achieves high accuracy in emotion classification tasks, demonstrating outstanding performance and generalization ability and providing new directions for future research in EEG signal recognition.

## 1. Introduction

### 1.1. Motivation

Emotions are fundamental to human behavior. Positive emotions help improve daily work efficiency, while negative emotions can lead to erroneous decisions. Emotions play a crucial role in people’s lives, profoundly impacting cognition, decision-making, and interpersonal interactions. Positive emotion (PE) affects cognitive processes, including executive function (i.e., inhibition, working memory, cognitive flexibility) [1]. For example, positive emotions often increase motivation and focus, making people more willing to engage in tasks and better able to focus. Conversely, negative emotions such as anxiety or depression may be distracting and reduce productivity. At the same time, moderate positive emotions can improve working memory performance and may promote more creative and flexible thinking.

In recent years, the application of artificial intelligence technology has indeed become increasingly widespread across various fields, and emotion recognition technology has emerged as a key technology in areas such as human-computer interaction, disease assessment, emotion detection, and healthcare. Specifically, it can be used to assist mental health professionals to more accurately assess and diagnose mood disorders such as depression and anxiety. In terms of user experience and human-computer interaction, product design can be optimized by analyzing users’ emotional responses. In the field of education, students’ emotional fluctuations in the learning process can be understood to help teachers adjust teaching methods and improve learning efficiency.

Over the past decades, there are two main approaches used to analyze and classify emotions: the locationist approach and the constructive approach [2]. The first approach assumes that each emotion has a specific brain structure and pattern. Studies of the constructionist approach use affective valence models or motivational direction models to deal with emotions. Affective valence means dividing emotions into positive and negative. Positive emotions include happiness, joy, excitement, and interest, while negative emotions include sadness, anger, disappointment, and anxiety. On the contrary, the motivational direction category has different characteristics. On the other hand, the constructionist model consists of valence-arousal-dominance (VAD) and activity-temperature-weight (VTT) models, where the VAD model is commonly used. Valence (positiveness-negativeness/pleasure-displeasure) refers to the emotional tendency conveyed by the vocabulary, with positive values indicating positive emotions, negative values indicating negative emotions, and zero indicating neutral emotions. Arousal (active-passive) represents the emotional intensity elicited by the vocabulary, with high values indicating high excitement or arousal, and low values indicating low excitement or calmness. Dominance (dominant-submissive) refers to the authority and control conveyed by vocabulary, where high values indicate high authority or control, while low values indicate low authority or control [3]. It can be considered that there are three primary colors in color theory, combining different numerical values to create a color. In VAD, valence, arousal, and dominance are three dimensions that assign a value to represent an emotion.

Emotion is a neurophysiological state that includes thoughts, feelings, excitement, pleasure, and personal behavioral responses [4]. Generally, human emotions can be detected in two ways: non-physiological signals and physiological signals [5]. Non-physiological signals include facial expressions, voice, language, gestures, etc. [6,7,8]. However, using non-physiological signals in emotion detection and classification may suffer from inaccurate recognition results because humans can produce false verbal intonations and facial expressions [9,10], and these technologies themselves are also fragile. Compared to non-physiological signals, physiological signals are harder to hide or disguise, making them more reliable. Physiological signals include electroencephalogram (EEG), electrooculography (EOG), electrocardiogram (ECG), electromyogram (EMG), galvanic skin response (GSR), skin temperature (ST), blood volume pulse (BVP), etc. EOG, ECG, EMG, and other physiological signals are usually indirect reactions caused by emotions, with lower recognition accuracy [11]. EEG, on the other hand, is generated by the central nervous system and directly reflects brain activity, making it possible to use EEG signals to reflect the working state of the human brain. Most researchers believe that EEG signals are more objective and accurate for studying human emotion recognition [12,13]. It has been confirmed by various psychophysiological studies that there are correlations between EEG signals and human emotions. Of course, it should also be noted that some signals are better for arousal (e.g., gsr), while others can be used for valence (EEG).

### 1.2. Related Work

Research on emotion recognition based on EEG signals mainly includes two parts: feature extraction and emotion classification models. EEG signals, as a kind of physiological signal with a low signal-to-noise ratio that is non-stationary and asymmetric, pose significant challenges for robust feature extraction and selection of emotional EEG signals [14]. Traditional methods of emotion recognition based on EEG signals mainly extract features in the time domain or frequency domain. For instance, Zhu et al. [15] used differential entropy (DE) as features and a support vector machine (SVM) for classification. Wagh et al. [16] utilized discrete wavelet transform (DWT) to decompose EEG signals in different frequency bands and employed SVM, k-nearest neighbors (KNN), and decision trees to classify emotional states on the SEED dataset. Liu et al. [17] adopted a method combining multi-channel EEG in the time domain with text features to recognize different human emotions, incorporating six time-domain statistical features into a single feature vector for emotion classification. Finally, the performance was evaluated on the DEAP dataset for physiological signal emotion analysis. Gannounid et al. [18]. proposed ZTWBES algorithm to identify time points, to select electrodes that successfully identify time points in advance, and to determine the relevant electrodes for each frequency band for each emotional state. Changes in brain activity compared with neutral emotional states were extracted as features.

However, the limitations of traditional electroencephalogram (EEG) analysis methods for emotion recognition have become increasingly apparent. These traditional methods have limited generalization ability and require extensive prior knowledge to obtain deep features of EEG signals. This results in significant time and energy consumption during the manual feature extraction process, greatly affecting the accuracy of emotion recognition [19].

In recent years, with the continuous development of artificial intelligence, deep learning has gradually gained popularity in various fields, attracting widespread attention from computer vision, speech recognition, natural language processing, biomedical signal processing, and other fields. Many researchers have successfully introduced deep learning methods into the field of EEG-based emotion recognition. Tripathi et al. [20] extracted features such as skewness and kurtosis from EEG signals in the DEAP dataset, which represents the benchmark for emotion classification research. Then, they used deep neural networks (DNNs) and convolutional neural networks (CNNs) to carry out binary and ternary classification for valence and arousal features, and the accuracy of this model has been greatly improved compared with previous studies.

Kim et al. [21] proposed a long short-term memory network considering the changes in emotions over time and applied an attention mechanism to allocate weights to the emotional states that appear at specific moments based on the peak–end rule in psychology. They used 32-channel EEG data from the DEAP database. Two-level (low and high) and three-level (low, middle, and high) classification experiments were performed on the valence and arousal emotion models. Wang et al. [22] proposed the concept of an electrode-frequency distribution map (EFDM) based on the short-time Fourier transform (STFT). They employed a deep convolutional neural network with residual blocks for automatic feature extraction and classification on the DEAP dataset. Cui et al. [23] designed an end-to-end regional asymmetric convolutional neural network (RACNN) for emotion recognition considering spatial information from neighboring and symmetric channels. Li et al. [24] proposed an emotion recognition method based on EEG combining multi-scale residual networks (MSRN) and meta-transfer learning (MTL) strategies and evaluated its performance using the DEAP and SEED datasets. Rajpoot et al. [25] proposed a subject-independent emotion recognition method, which uses an unsupervised Long Short-Term Memory (LSTM) with channel-wise attention autoencoder.

### 1.3. Contribution

Although many studies in the literature have proposed various deep learning methods and models for EEG-based emotion recognition and classification, there are still many unresolved issues, such as the following: (1) The original EEG signals already contain a lot of information, but due to the nonlinear nature of these signals, it is difficult to directly extract valuable features. (2) How to design the structure or framework of the EEG emotion decoding model that can fully explore high-level abstract features while maintaining high accuracy and robustness. (3) EEG data are composed of multiple channels; how to find the best channels for feature extraction without manual selection. To tackle the aforementioned challenges, this study introduces a hybrid deep learning model named DACB, which integrates a dual attention mechanism, a convolutional neural network, and a bidirectional long short-term memory network for effective emotion classification.

The primary contributions of this paper are outlined as follows: (1) The proposed model in this study utilizes only the original EEG signals, eliminating the need for any time-frequency domain transformation for feature extraction. This simplifies its portability and integration into brain-computer interface devices. (2) While prior studies predominantly emphasized spatial feature extraction, temporal sequence features play a pivotal role in EEG signals. Hence, this paper introduces a bidirectional long short-term memory network to enhance the model’s capability in capturing temporal features. (3) A one-dimensional Squeeze-and-Excitation (SE) network attention mechanism alongside a dot product attention mechanism was introduced. The former enables the model to learn the significance of various channel features, while the latter teaches it the importance of spatial and temporal features. (4) By applying the model to two-dimensional datasets, this paper evaluates its performance across both the discrete emotion model and the three dimensions of the multidimensional emotion space model (valence, arousal, and dominance). This comprehensive testing further validates the model’s generalization capabilities by using EEG signals.

The rest of the study is structured as follows: Section 2 details the methods presented in this paper and provides a description of the dataset and experimental setup. Section 3 describes the basic process of the experiment and compares the experimental results with other methods. Finally, Section 4 summarizes the main conclusions of this study.

## 2. Materials and Methods

### 2.1. The Structure of the DACB Model

**Figure 1:**
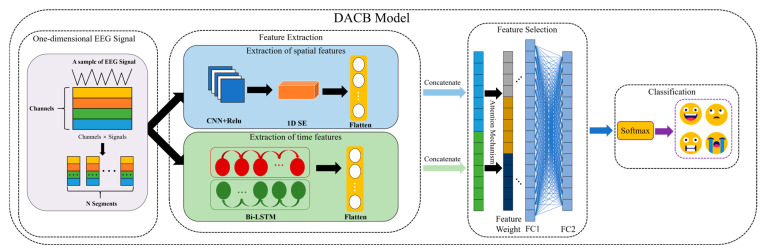
DACB structure.

Figure 1 illustrates the basic structure of the DACB model. As shown in Figure 1, the DACB model consists of four components: EEG input for emotion recognition, feature extraction, feature selection, and classification. Firstly, the raw one-dimensional EEG signals are normalized and then simultaneously fed into the feature extraction module. The feature extraction has two parallel channels, including spatial features and temporal features.

As shown in Figure 1, the DACB structure includes a spatial feature extraction module, which consists of a convolutional layer with a ReLU activation function, a max pooling layer, an SE attention mechanism module, and a flatten layer. The convolutional layer primarily captures the interaction information between spatial physiological signals and feature channels. The ReLU activation function is simple and efficient, effectively alleviating the problems of gradient disappearance and explosion, and has been widely used in recent years. The SE attention mechanism learns the importance of each channel in the feature mapping from the channel dimension data using a two-layer neural network and then multiplies it by the original channel data. The temporal feature extraction module is composed of two layers: Bidirectional Long Short Term Memory network (Bi-LSTM) and Flatten. By considering both forward and backward time series information simultaneously along the temporal dimension, Bi-LSTM can achieve more comprehensive and accurate predictions.

The EEG signals are processed through a spatial feature extraction module, where the data’s spatial features are obtained using CNN. Then, the SE attention mechanism is applied to further derive the weight distribution across various channels. Meanwhile, the Bi-LSTM extracts temporal features from the EEG time series signals. A flatten layer concatenates the features of two different dimensions, and, finally, a fully connected layer integrates these features to output classification results. This dual-channel model allows for a more in-depth analysis of emotional features from both temporal and spatial perspectives, thereby enhancing the accuracy of the prediction results. The DACB architecture parameters are shown in Table 1.

### 2.2. Convolutional Neural Network

In recent years, Convolutional Neural Networks (CNNs) have achieved significant performance in various challenging tasks such as computer vision, speech recognition, and natural language processing (NLP). For instance, CNNs can outperform humans in some difficult visual tasks [26]. Moreover, they are not limited to specific types of input data but excel in various fields, including but not limited to signal processing for brain-computer interfaces (BCI) and biomedical signal processing [27,28].

CNNs have emerged as the most prevalent deep-learning-based network model. Contrary to traditional machine learning algorithms, CNNs eliminate the need for manual feature design. Instead, they leverages local receptive fields created by convolutional kernels to automatically extract abstract features from raw data for classification, thereby preventing the loss of valuable information. Unlike the traditional framework that separates feature learning and classification into distinct steps, CNNs are capable of learning features and performing classification simultaneously through multiple layers of neural networks. A standard CNN comprises five layers: an input layer, a convolutional layer, an activation layer, a pooling layer, and a fully connected layer. Typically, a comprehensive CNN architecture consists of one or more such blocks, culminating in a classifier layer. Given a 2D or 3D array as input data *X* and a CNN with n computational blocks, the output of the convolutional layer in the *L*-th block can be computed using the following formula.

(1) $$f_{L(x)}=\sum_{i=1}^{L}\left(X_{i}*w_{i}+b_{i}\right)$$

Here, *X_i_*, *w_i_*, and *b_i_* represent the input, weight, and bias, respectively, with ∗ denoting the convolution operation. Once the activation and pooling layers have been computed and processed accordingly, the output of the convolutional layer serves as the input for the subsequent block. The output of the final convolutional layer is then fed consecutively into the following fully connected layer and classifier layer. The entire network undergoes training through the backpropagation of a supervised loss function.

### 2.3. Bidirectional Long Short-Term Memory Network

**Figure 2:**
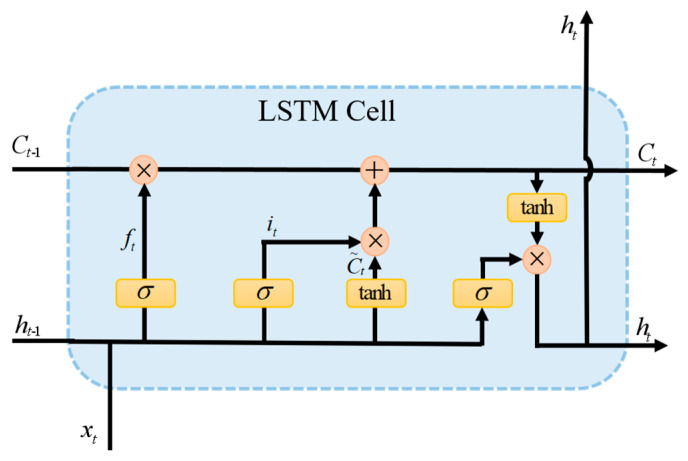
LSTM cell.

There exists a specific temporal dependency among the sequential signals within the emotional electroencephalogram. To better capture the temporal dependency features of the time series, this paper incorporates Bidirectional Long Short-Term Memory (Bi-LSTM). Bi-LSTM not only processes the contextual information but also incorporates the content of future contexts, thereby enhancing the model’s accuracy. Bi-LSTM consists of two LSTMS. The LSTM network comprises “gates” with distinct functions and operations. Figure 2 illustrates the LSTM cell unit and its internal structure.

The forget gate determines which information should be discarded or retained, as expressed in the following formula.

(2) $$f_{t}=\sigma (w_{f}\times [h_{t-1},x_{t}]+b_{f})$$

The aim of updating the input gate and output gate is to ascertain whether to substitute the memory cell with a candidate value and produce the activation segment of the current time step. This process can be further represented as follows:

(3) $$i_{t}=\sigma (w_{i}\times [h_{t-1},x_{t}]+b_{i})$$

(4) $$o_{t}=\sigma (w_{o}\times [h_{t-1},x_{t}]+b_{o})$$

The behavior of the LSTM unit is determined by the following formula.

(5) $${\tilde{C}}_{t}=\tanh(w_{c}\times [h_{t-1},x_{t}]+b_{c})$$

(6) $$C_{t}=f_{t}\times C_{t-1}+i_{t}\times {\tilde{C}}_{t}$$

(7) $$h_{t}=o_{t}\times \tanh(C_{t})$$

In the formula, *σ* denotes the sigmoid activation function, while tanh represents the hyperbolic tangent activation function. *w_f_*, *w_i_*, and *w_o_* stand for weight matrices, *x_t_* represents the input vector, *h_t__−_*_1_ signifies the previous hidden state, and *b_i_*, *b_o_*, and *b_c_* represent the bias vector.

In Bi-LSTM, the feature data obtained at time *t* contain both past and future information. Compared to a single LSTM structure, Bi-LSTM is more effective in extracting EEG signal features. Bi-LSTM can utilize both early and late sequence information, aiding in uncovering deep brain information from long EEG signal sequences, with its performance surpassing that of a unidirectional LSTM [29].

(8) $$h_{t}=\sigma (W_{h}\times [\vec{h_{t}},\overset{\leftarrow }{h_{t}}]+b_{h})$$

$\vec{h_{t}}$ represents the current state in the forward direction, and $\overset{\leftarrow }{h_{t}}$ represents the current state in the backward direction. *W_h_* and *b_h_* represent the weight matrix and bias vector.

### 2.4. 1D SE-Block

The 2D SE attention mechanism, proposed by Hu et al. [30], secured the top spot in the ILSVRC 2017 classification task, demonstrating that the integration of SE blocks can bolster network performance. The SE attention mechanism, which operates on a channel-based approach, elevates attention along the channel dimension, emphasizing squeeze and excitation. This mechanism primarily identifies the attention level of each channel within the feature map, assigning an attention weight to each feature channel accordingly. This allows the convolutional network to prioritize these feature channels, thereby emphasizing the channels in the feature map that are pertinent to the current task while suppressing those that are less relevant.

**Figure 3:**
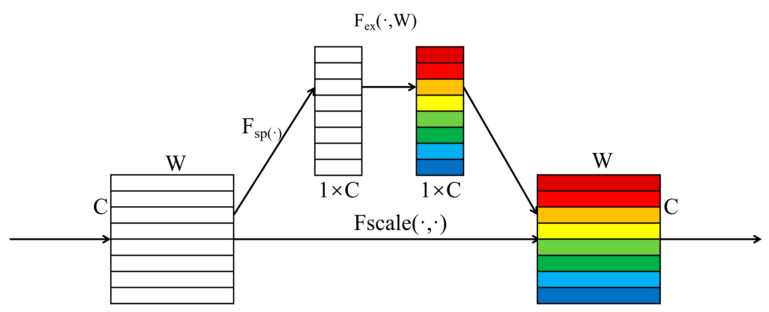
1D SE-block.

Hence, this paper designs an SE block for one-dimensional data, as depicted in Figure 3. As shown in Figure 3, this one-dimensional SE module can deliberately amplify or attenuate specific channels by leveraging global information, thus enhancing the network’s performance. When compared to the straightforward usage of a single fully connected layer in the excitation operation, the benefit of employing two fully connected layers lies in their increased nonlinearity, enabling a superior fit for the intricate correlations among channels. Consequently, the one-dimensional SE block is well-suited for applications in electroencephalogram signal classification.

The one-dimensional SE block comprises two integral components: squeeze and excitation. Initially, the squeeze operation quantifies each feature channel into a corresponding real number (*Z_c_*) by employing a one-dimensional global average pooling operation. The formula for computing *Z_c_* is as follows [30]:

(9) $$Z_{c}=F_{sq}(u_{c})=\frac{1}{W}\sum_{i=1}^{W}u_{c}(i)$$

*W* is the data length of a channel, and *u_c_* is the feature channel, *Z_c_* ∈ *R^c^*. Excitation generates the weight values *S_c_* [30] for each feature channel, calculated as follows:

(10) $$S_{c}=F_{ex}(z_{c},W)=\sigma (g(z_{c},W))=\sigma (W_{2}\delta (W_{1}z_{C}))$$

*σ* and *δ* are the Sigmoid and ELU activation functions. *W*_1_ is the first fully connected layer (dimensionality reduction layer), *W*_1_ ∈$\;R^{\frac{c}{r}\times c}$, controlled by the reduction ratio (R). *W*_2_ is the second fully connected layer (dimensionality increase layer), *W*_2_ ∈ $R^{\frac{c}{r}\times c}$.

Finally, multiply the weight values *s_c_* of the feature channel by the original channel *u_c_* to obtain the output of the one-dimensional SE block [31], as shown below.

(11) $$F_{scale}(u_{c},s_{c})=u_{c}s_{c}$$

### 2.5. Dot Multiplier Attention Mechanism

Common attention mechanisms include dot product attention, scaled attention, spatial attention, and others. In this article, apart from utilizing the SE attention mechanism, we also incorporate dot product attention, which significantly improves the model’s accuracy, reduces computation time, and simplifies algorithm complexity. Within the dot product attention mechanism, attention scores are derived by computing the dot product between query and key vectors. Specifically, for a designated query vector *Q* and key vector *K*, the formula for calculating dot product attention is as follows: attention score = *Q*·*K*.

**Figure 4:**
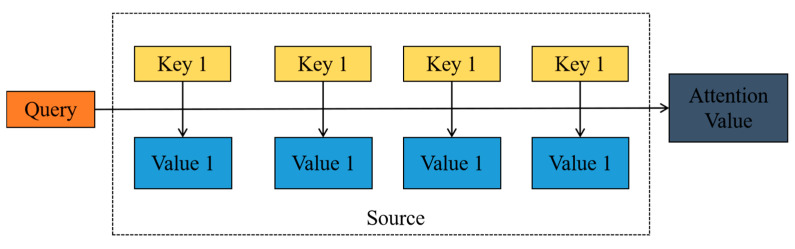
Dot multiplier attention mechanism.

The dot product attention mechanism stands out for its efficient and straightforward calculation. It generates attention scores by directly computing the dot product between query and key vectors, eliminating the need for extra parameters or complex computational steps. Take the dot product attention mechanism as an example; its role is to assign weights to the expressions of hidden layer vectors outputted by the model. By integrating the attention mechanism into the feature extraction model, features that have a more significant impact on the output variables can be prioritized, thereby improving the accuracy of the method. Figure 4 illustrates the attention mechanism for point products.

The dot product attention comprises three integral components: the key matrix *K*, the value matrix *V*, and the query vector *q* [32].

First, Equation (12) defines the method for calculating the key matrix.

(12) $$K=\tanh(VW^{a})$$

In this formula, *W^a^* is a randomly initialized weight matrix, *V* is the original input vector, and *K* is the computed key vector. Using the hyperbolic tangent function tanh ensures that the values of the key vector are within the range of −1 to 1.

(13) $$d=soft\max(qK^{T})$$

Next, Equation (13) describes how to calculate the attention weights (also known as the normalized probability vector *d*). Here, *q* is the query vector, and *K^T^* is the transpose of the key vector. The *soft* max function converts the computed attention scores into a probability distribution format, ensuring that the sum of all scores equals 1.

(14) a=dV

Finally, Equation (14) defines how to apply the attention weights to obtain the attention vector *a*. Here, *d* is the previously calculated attention weight vector, and *V* is the original value vector. By multiplying the attention weights with the value vector, a new vector can be obtained that reflects the importance of various positions in the original vector.

### 2.6. Dataset

Our experiments were carried out on two widely used datasets: the Database for Emotion Analysis using Physiological Signals (SEED-IV) [33,34,35] (https://bcmi.sjtu.edu.cn/~seed/index.html, accessed on 18 December 2024) and the Database for Emotion Recognition utilizing EEG and ECG signals (DREAMER) [36,37] (https://zenodo.org/records/546113, accessed on 18 December 2024).

The movie clips used for both datasets are shown in Table 2 and Table 3.

The Shanghai JiaoTong University Emotional EEG Dataset (SEED-IV) is generously provided by Professor Lu from the BCMI Lab at Shanghai Jiao Tong University. SEED-IV comprises EEG recordings from 15 participants, with each participant undergoing three separate experiments conducted on different days. Each experiment encompassed 24 movie clips. During each experiment, participants watched a movie clip while their EEG signals and eye movements were meticulously captured from 62 channels, utilizing the ESI neuroimaging system alongside SMI eye-tracking glasses. The objective of these experiments was to evoke feelings of happiness, sadness, fear, or neutrality. The selection criteria for movie clips were as follows: (1) the duration of each experiment was kept brief to prevent participant fatigue, (2) the videos were designed to be self-explanatory, and (3) the videos were intended to evoke a singular target emotion.

DREAMER is a multimodal physiological dataset for emotional computing, collected by Katsigiannis et al. The experimental data comprise multi-channel EEG signals gathered from 23 healthy subjects (9 females and 14 males). In this database, emotions were elicited in each subject by presenting 18 movie clips. Each movie clip encompassed nine emotion categories: anger, fear, calm, amusement, sadness, surprise, disgust, happiness, and excitement. The duration of each movie clip varied from 65 to 393 s, averaging 199 s. EEG signal acquisition utilized 14 channels (adhering to the standard 10–20 electrode system) with a sampling rate of 128 Hz, while ECG signal acquisition employed a sampling rate of 256 Hz. Following the viewing of each movie clip, subjects self-assessed their valence, arousal, and dominance using a two-dimensional emotion space model, scoring from 1 to 5 as the outcome classification labels. Prior to the commencement of each clip, subjects were instructed to watch a neutral video to assist in adjusting their emotions to a neutral state.

To test the stability and portability of the model in emotion recognition, experiments were conducted on the SEED-IV and DREAMER datasets. The parameter of the original signal is channel × time, the number of channels in the SEED-IV dataset is 62, and the number of channels in DREAMER is 14. Due to the large amount of data, 1000 consecutive datasets were extracted from each person in the experiment in the middle of each video segment, resulting in 360,000 for SEED and SEED-IV (15 people × 24 videos × 1000 EEG data) and 1,242,000 for 23 people × 18 videos × 1000 EEG data × three labels (valence, arousal, and dominance).

### 2.7. Experimental Setup

In this experiment, due to the significant temporal correlation of each individual’s EEG data, using the same person’s EEG data as both training and testing sets may lead to biased and unreasonable results. Therefore, to ensure more reasonable and accurate results, this study divides the experiments of the SEED-IV dataset into approximately 80% (training set of 12 individuals) and approximately 20% (testing set of 3 individuals) and similarly divides the experiments of the DREAMER dataset into approximately 80% (training set of 18 individuals) and approximately 20% (testing set of 5 individuals). The experiment was conducted with the Adam optimizer for 150 training epochs, with a batch size of 1024.

To further validate the performance of the model, this paper also conducted a 10-fold cross-validation experiment. Ten-fold cross-validation involves dividing the dataset into 10 parts, with 9 parts of EEG signals used for the training set and the remaining 1 part as the test set, and so on, repeating this process 10 times. Next, the experimental results of the test set are saved, and the average value is used as the model evaluation criterion. This strategy can promote the model to learn samples from multiple aspects, avoiding the model becoming stuck in local extreme values. Ensuring the same data distribution between the training and test sets, all pre-training data are set with the same random seed, randomly shuffled, and passed to the network model. This paper implemented and modeled DACB and other comparative network models using the same parameter settings on GeForce RTX 4090 (Colorful, Xi’an, China). The training parameters of the DACB model are shown in Table 4.

### 2.8. Evaluation Index

The evaluation criteria used in the paper include accuracy, precision, recall rate, F1-score, and Matthew’s correlation coefficient to assess the model’s performance. Accuracy measures the proportion of correctly predicted samples among all samples. Precision is the proportion of true positive samples among all positive samples predicted by the classifier. Recall rate measures the proportion of correctly predicted positive samples among all true positive samples. F1-score is the harmonic mean of precision and recall. MCC is calculated based on the confusion matrix, considering true positive, true negative, false positive, false negative, etc., providing a comprehensive evaluation of the model’s performance. Specifically, the range of MCC values is [−1, 1], where 1 indicates perfect predictive ability, 0 indicates random prediction or no predictive ability, and −1 indicates completely opposite predictive ability. When MCC is a positive number, it represents a higher likelihood of the model’s predicted results matching the actual situation; when MCC is a negative number, it represents a higher likelihood of the model’s predicted results being opposite to the actual situation. The definitions of accuracy, precision, recall rate, F1-score, and Matthew’s correlation coefficient are as follows:

(15) $$accuracy=\frac{TP+TN}{TP+TN+FP+FN}$$

(16) $$precision=\frac{TP}{TP+FP}$$

(17) $$recall=\frac{TP}{TP+FN}$$

(18) $$F1-score=\frac{2}{\frac{1}{precision}+\frac{1}{recall}}$$

(19) $$MCC=\frac{TP\times TN-FP\times FN}{\sqrt{\left(TP+FP)(TP+FN)(TN+FP)(TN+FN\right)}}$$

Among them, *TP* represents true positive samples; *TN* represents true negative samples; *FP* represents false positive samples, that is, incorrectly predicted as positive samples; and *FN* represents false negative samples, that is, incorrectly predicted as negative samples positive samples.

## 3. Results

### 3.1. Single-Test Result of DACB Model

To validate the proposed DACB model’s classification performance in EEG-based emotion detection, this study compares the model with Convolutional Neural Network-Recurrent Neural Network (CNN-RNN), Convolutional Neural Network-Long Short-Term Memory (CNN-LSTM), Convolutional Neural Network-Bidirectional Long Short-Term Memory (CNN-Bi-LSTM), 1D Convolutional Autoencoder (1D CAE), and 1D InceptionV1. 1D CAE consists of two convolutional layers replacing fully connected layers, with input symbols downsampled to provide smaller-dimension latent representations. On the other hand, 1D InceptionV1 replaces the 2D convolutional kernels of InceptionV1 with 1D convolutional kernels. In addition to comparing this model with other deep learning models, we also compare it with three popular traditional machine learning types: Adaboost, Bayes, and XGBoost. Traditional machine learning methods have also found widespread applications in various computer fields. Traditional machine learning typically requires manual feature engineering, extracting meaningful features from raw data. These methods are simple, effective, and interpretable for particularly straightforward tasks. In contrast, deep learning can automatically learn features through multi-layer neural networks, obtaining more sophisticated representations from raw data.

The DACB model was validated on the SEED-IV and DREAMER datasets, as presented in Table 5, Table 6, Table 7 and Table 8. The SEED-IV dataset comprises four distinct classes of labels, happiness, sadness, fear, and neutrality, representing various emotional states. On the other hand, the DREAMER dataset employs a two-dimensional emotional space model for self-assessment of valence, arousal, and dominance, with result classification labels ranging from 1 to 5. The DABC model is trained and tested separately in valence, arousal, and dominance.

In the SEED-IV dataset, the DACB model proposed in this paper performed the best in the four-classification task, with an accuracy of 99.96%, precision of 99.96%, recall of 99.96%, F1-score of 99.96%, and MCC of 99.95%. The CNN-Bi-LSTM model followed closely behind DACB, with an accuracy of 99.96%, precision of 99.96%, recall of 99.96%, F1-score of 99.96%, and MCC of 99.95%. Bayes showed the worst results, with an accuracy of 26.10%, precision of 30.44%, recall of 26.10%, F1-score of 17.39%, and MCC of 2.46%.

In the valence classification task on the DREAMER dataset, the DACB model achieved an accuracy of 87.52%, precision of 87.74%, recall of 87.41%, F1-score of 87.52%, and MCC of 84.28%. In the arousal classification task, the DACB model achieved an accuracy of 90.06%, precision of 90.15%, recall of 89.40%, F1-score of 89.76%, and MCC of 86.75%. In the dominance classification task, the DACB model achieved an accuracy of 89.05%, precision of 89.93%, recall of 88.69%, F1-score of 89.28%, and MCC of 85.24%. The DACB model performed the best in all three classification tasks on the DREAMER dataset, while the Bayesian method performed the worst. The results indicate that the proposed DACB model outperforms other models because its parallel structure can extract spatial-temporal features of input signals simultaneously, thereby improving model accuracy.

### 3.2. Ten-Fold Cross Validation Result of DACB Model

**Figure 5:**
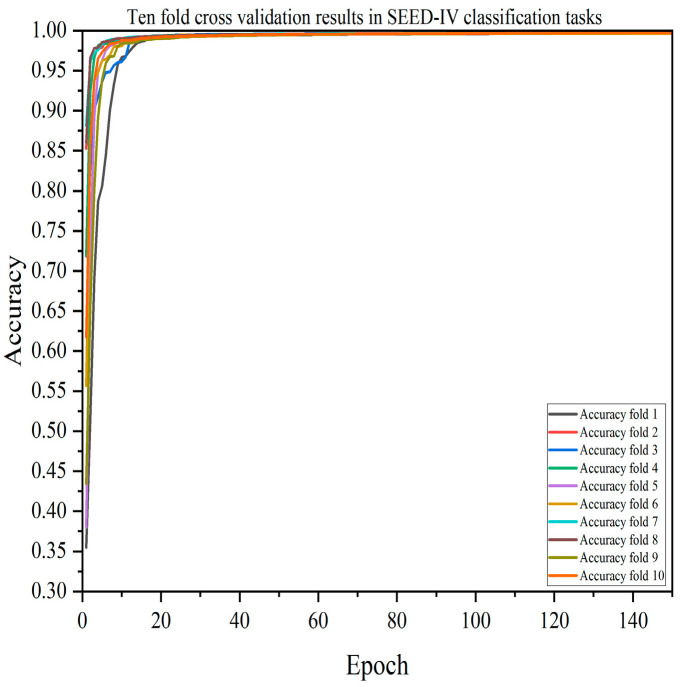
Accuracy of the DACB model on the SEED-IV dataset based on 10-fold cross-validation.

**Figure 6:**
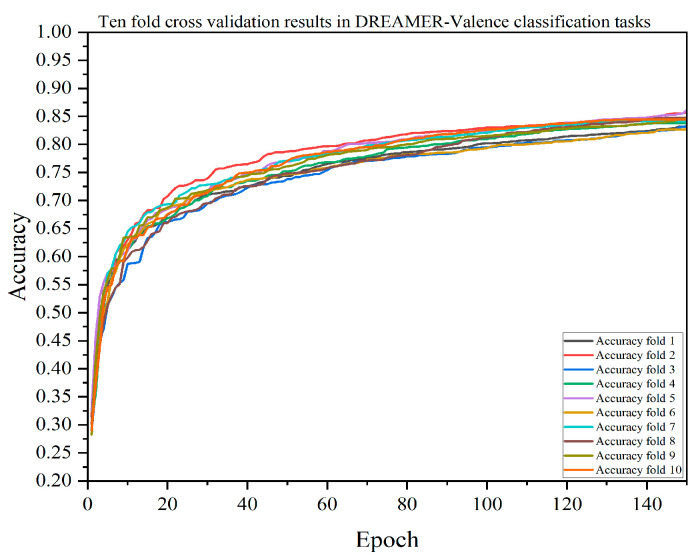
Accuracy of the DACB model on the DREAMER-Valence dataset based on 10-fold cross-validation.

**Figure 7:**
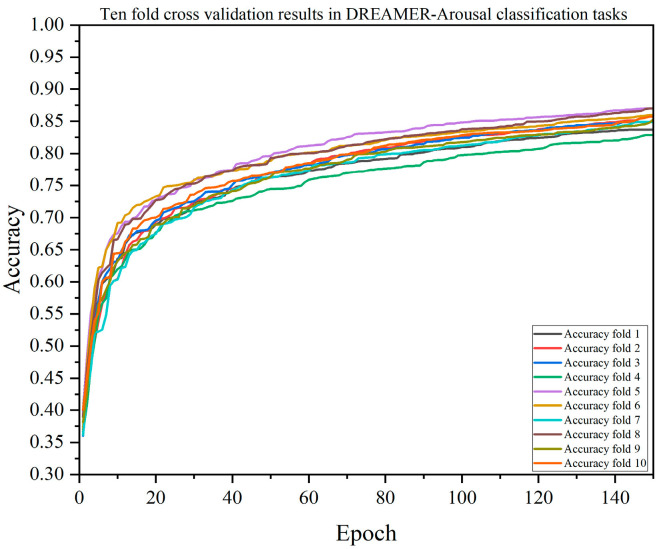
Accuracy of the DACB model based on 10-fold cross-validation on the DREAMER-Arousal dataset.

**Figure 8:**
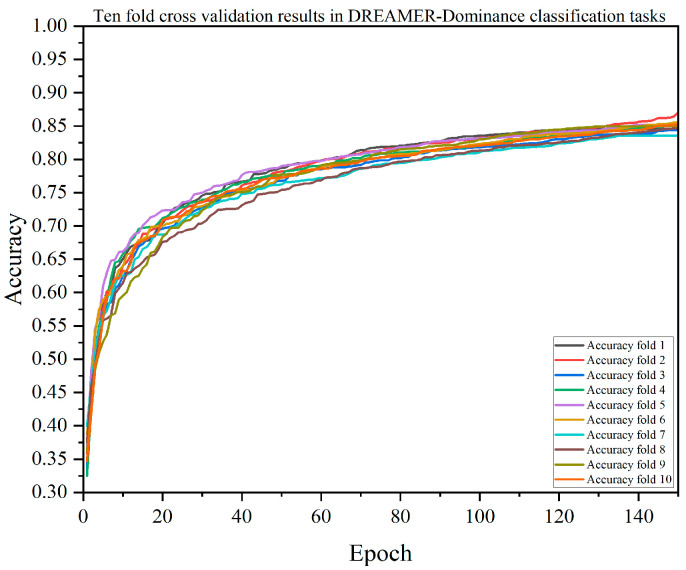
The accuracy of the DACB model on the DREAMER-Dominance dataset based on 10-fold cross-validation.

A single-model test verification may have randomness and uncertainty, so this paper also verified the performance of each model through 10-fold cross-validation. The results of the DACB model verified by 10-fold cross-validation are shown in Figure 5, Figure 6, Figure 7 and Figure 8. Table 9, Table 10, Table 11 and Table 12 summarize the average results of 10-fold cross-validation.

In the SEED-IV dataset, as shown in Table 9, the DACB model proposed in this paper still performs the best in the four-classification task, with an accuracy of 99.73%, precision of 99.72%, recall of 99.72%, F1-score of 99.72%, and MCC of 99.64%. Similarly, in 10-fold cross-validation, the CNN-Bi-LSTM model is only second to DACB, with an accuracy of 92.87%, precision of 92.89%, recall of 92.87%, F1-score of 92.87%, and MCC of 90.50%.

In the valence classification task on the DREAMER dataset, as shown in Table 10, the accuracy of the DACB model is 84.26%, precision is 84.39%, recall is 84.03%, F1-score is 84.18%, and MCC is 80.12%. The accuracy of the XGBoost model is 75.90%, precision is 77.08%, recall is 75.15%, F1-score is 75.89%, and MCC is 69.48%. In the arousal classification task, as shown in Table 11, the accuracy of the DACB model is 85.40%, precision is 85.54%, recall is 84.63%, F1-score is 85.04%, and MCC is 80.49%. The accuracy of the XGBoost model is 76.96%, precision is 81.75%, recall is 73.75%, F1-score is 76.89%, and MCC is 68.97%. In the dominance classification task, as shown in Table 12, the accuracy of the DACB model is 77.56%, precision is 81.58%, recall is 84.47%, F1-score is 85.10%, and MCC is 79.85%. The accuracy of the XGBoost model is 77.56%, precision is 81.58%, recall is 76.03%, F1-score is 78.35%, and MCC is 69.79%.

In two datasets, among the traditional three machine learning models, XGBoost exhibits better performance, with its overall performance second only to the DCBA model proposed in this paper. This is likely because the XGBoost algorithm is an ensemble learning method that enhances model accuracy and stability by constructing multiple decision trees. Among the five deep learning models, the CNN-Bi-LSTM model performs well on the SEED-IV dataset but shows a decline in performance on the DREAMER dataset, indicating poor generalization ability of the model. From the results of single validation and 10-fold cross-validation, it can be seen that the model in this paper performs the best among all comparison models, indicating the robustness of the DACB model in EEG-based emotion classification.

### 3.3. Ablation Experiment

To demonstrate and validate the benefits of concurrently extracting spatiotemporal information within a multi-channel model, this study also conducted ablation experiments on the SEED-IV and DREAMER datasets. Block1 eliminated the temporal feature module, while Block2 removed the spatial feature module, with all other modules remaining unchanged. The results of the ablation experiments are presented in Table 13 and Table 14, revealing that the single-channel spatial feature module performs inferiorly to the DACB model. In the SEED-IV dataset, Block2 outperforms Block1, indicating that temporal features are relatively important. It is noteworthy that, in the dreamer dataset, Block1 and Block2 show a performance difference of approximately 50% compared to our model, highlighting the superiority of the DACB model in combining spatial and temporal features for emotion recognition. This also demonstrates that relying solely on spatial or temporal features does not yield satisfactory experimental results, further validating the effectiveness of the proposed method.

### 3.4. Feature Visualization

**Figure 9:**
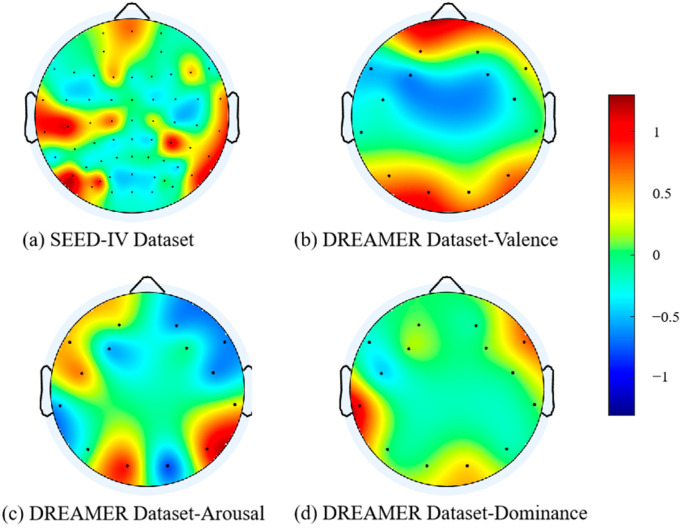
Visualization of electrode channel contribution.

This article adopts the SE (Squeeze-and-Excitation) attention mechanism, which enhances the model’s expressiveness by assigning different weights to different channels. To improve the model’s interpretability, the channel weights are visualized. Figure 9 presents the visualization results of electrode channel contributions: (a) corresponds to the analysis results of the SEED-IV dataset, while (b), (c), and (d) show the analysis results for the three emotional dimensions of valence, arousal, and dominance in the DREAMER dataset. The visual attention score is not calculated by the softmax function, so there is a negative number. Emotion is a complex phenomenon involving various cognitive processing mechanisms, and these processes are thought to result from the interaction of bottom-up and top-down information processing pathways [38,39]. These cognitive mechanisms exhibit significant neural activity in the parietal, temporal-parietal, and occipital regions of the brain [40,41,42]. The distribution of electrode channel feature weights extracted from EEG (electroencephalogram) signals using the SE attention mechanism aligns with these known brain cognitive functional areas, further validating the interpretability and effectiveness of the model proposed in this study.

## 4. Conclusions

This study introduces a multi-channel automatic emotion electroencephalogram (EEG) signal classification method, the DACB model. This proposed model incorporates various strategies to enhance accuracy. The proposed DACB model, a multi-channel spatiotemporal feature fusion network, achieves impressive classification accuracies of 99.73%, 84.26%, 85.40%, and 85.02% across four classification tasks in the SEED-IV and DREAMER datasets. The DACB model effectively captures spatial features through a one-dimensional SE module, extracts temporal features using a bidirectional long short-term memory network tailored for EEG as a time series signal, and selects these critical features via a dot product attention mechanism. Testing results on two distinct open-source EEG emotion datasets demonstrate the remarkable capabilities of the proposed deep learning model, which can learn features and classify stages without relying on manual feature extraction. This model holds promise for designing efficient and accurate real-time brain-computer interface frameworks. In future work, we aim to further improve the DACB model, such as exploring how DACB methods can enhance artifact removal or detect specific pathological patterns in EEG, so as to improve the accuracy of EEG recognition and classification and reduce the computing overhead of the model to facilitate deployment in the usability testing framework of emotion detection based on EEG signals [43,44]. In addition, we plan to incorporate multi-modal data, including EMG, ECG, or MRI, to further improve the generalization of the model.

## Figures and Tables

**Table 1:** Parameters of the DACB architecture.

Module	Type	Number
Block1	Conv1d	64
	Flatten	1
Block2	Bi-LSTM	32
	Flatten	1
FC1	Dense	64
FC2	Dense	32
FC3	Softmax	1

**Table 2:** SEED-IV dataset movie snippets.

Serial No.	Film Clips’ Sources	Emotion Label	Start Time Point	End Time Point
01	Black Keys	sad	42:32	45:41
02	The Eye 3	fear	49:25:00	51:00:00
03	Rob-B-Hood	happy	41:07:00	45:06
04	A Bite of China	neutral	30:29	32:48
05	The Child’s Eye	fear	41:00	42:37
06	A Bite of China	neutral	5:19	8:05
07	A Bite of China	neutral	24:42	26:41
08	Very Happy	sad	17:09	21:13
09	A Bite of China	neutral	31:18	33:44
10	A Wedding Invitation	sad	1:34:04	1:38:50
11	Bunshinsaba II	fear	42:24	43:33
12	Dearest	sad	1:31:08	1:33:29
13	Aftershock	sad	20:13	24:14
14	Foster Father	sad	24:29	27:10
15	Bunshinsaba III	fear	1:04:52	1:09:49
16	Promo for applying the Olympic Winter Games	happy	0:00	2:54
17	Hungry Ghost Ritual	fear	45:07	46:48
18	Hungry Ghost Ritual	fear	1:10:21	1:13:33
19	Very Happy	happy	34:30	37:15
20	You are my life more complete	happy	39:32	40:44
21	A Bite of China	neutral	18:59	20:56
22	Hear Me	happy	1:33:27	96:10
23	A Bite of China	neutral	16:28	19:24
24	Very Happy	happy	12:48	15:31

**Table 3:** DREAMER dataset movie snippets.

Serial No.	Film Clips’ Sources	Valence	Arousal	Dominance
01	Searching for Bobby Fischer	3.17 ± 0.72	2.26 ± 0.75	2.09 ± 0.73
02	D.O.A.	3.04 ± 0.88	3.00 ± 1.00	2.70 ± 0.88
03	The Hangover	4.57 ± 0.73	3.83 ± 0.83	3.83 ± 0.72
04	The Ring	2.04 ± 1.02	4.26 ± 0.69	4.13 ±0.87
05	300	3.22 ± 1.17	3.70 ± 0.70	3.52 ± 0.95
06	National Lampoon’s VanWilder	2.70 ±1.55	3.83 ± 0.83	4.04 ± 0.98
07	Wall-E	4.52 ± 0.59	3.17 ± 0.98	3.57 ± 0.99
08	Crash	1.35 ± 0.65	3.96 ± 0.77	4.35 ± 0.65
09	My Girl	1.39 ± 0.66	3.00 ± 1.09	3.48 ± 0.95
10	The Fly	2.17 ± 1.15	3.30 ± 1.02	3.61 ± 0.89
11	Pride and Prejudice	3.96 ± 0.64	1.96 ± 0.82	2.61 ± 0.89
12	Modern Times	3.96 ± 0.56	2.61 ± 0.89	2.70 ± 0.82
13	Remember the Titans	4.39 ± 0.66	3.70 ± 0.97	3.74 ± 0.96
14	Gentleman’s Agreement	2.35 ± 0.65	2.22 ± 0.85	2.39 ± 0.72
15	Psycho	2.48 ± 0.85	3.09 ± 1.00	3.22 ± 0.9
16	The Bourne Identity	3.65 ± 0.65	3.35 ± 1.07	3.26 ± 1.14
17	The Shawshank Redemption	1.52 ± 0.59	3.00 ± 0.74	3.96 ± 0.77
18	The Departed	2.65 ± 0.78	3.91 ± 0.85	3.57 ± 1.04

**Table 4:** DACB model training parameters.

Parameters	Number
epoch number	150
learning rate	0.001
batch size	1024
optimizer	Adam
loss function	categorical_crossentropy
Random seed	42

**Table 5:** The performance of the DACB model and other models on SEED-IV of task test sets.

Methods	Accuracy (%)	Precision (%)	Recall (%)	F1-Score (%)	MCC (%)
CNN-RNN	61.53	62.94	61.53	60.72	49.50
CNN-Bi-LSTM	96.15	96.16	96.15	96.15	94.88
1D CAE	90.28	90.28	90.28	90.28	87.05
1D InceptionV1	79.28	79.73	79.28	79.33	72.49
Adaboost	37.49	37.52	37.49	37.41	16.69
Bayes	26.10	30.44	26.10	17.39	2.46
XGBoost	87.25	87.35	87.24	87.26	83.02
DACB	99.96	99.96	99.96	99.96	99.95

**Table 6:** The performance of the DACB and other models on DREAMER-Valence of task test sets.

Methods	Accuracy (%)	Precision (%)	Recall (%)	F1-Score (%)	MCC (%)
CNN-RNN	58.25	59.13	58.25	58.46	47.52
CNN-Bi-LSTM	75.68	76.16	75.29	75.65	69.31
1D CAE	67.38	67.45	67.24	67.24	58.89
1D InceptionV1	34.82	39.30	32.29	31.93	16.82
Adaboost	34.66	34.63	32.70	32.64	16.76
Bayes	22.53	23.94	22.28	15.96	3.96
XGBoost	81.16	82.08	80.86	81.35	76.24
DACB	87.52	87.74	87.41	87.52	84.28

**Table 7:** The performance of DACB and other models on DREAMER-Arousal of task test sets.

Methods	Accuracy (%)	Precision (%)	Recall (%)	F1-Score (%)	MCC (%)
CNN-RNN	63.38	66.64	58.71	61.44	50.56
CNN-Bi-LSTM	76.47	77.89	74.50	76.00	68.49
1D CAE	71.38	74.15	68.34	70.68	61.56
1D InceptionV1	40.59	49.41	31.42	32.20	17.85
Adaboost	38.69	37.20	30.62	31.22	15.72
Bayes	15.26	25.45	25.29	15.54	4.78
XGBoost	81.95	85.29	80.56	82.62	75.81
DACB	90.06	90.15	89.40	89.76	86.75

**Table 8:** The performance of DACB model and other models on DREAMER-Dominance of task test sets.

Methods	Accuracy (%)	Precision (%)	Recall (%)	F1-Score (%)	MCC (%)
CNN-RNN	65.20	67.11	62.17	64.06	52.73
CNN-Bi-LSTM	73.21	76.76	70.68	73.01	63.67
1D CAE	70.46	71.91	71.04	70.94	60.56
1D InceptionV1	40.21	47.56	29.78	29.66	15.74
Adaboost	37.30	33.68	29.27	29.69	12.86
Bayes	21.36	25.75	26.19	18.09	6.37
XGBoost	82.33	85.88	82.27	83.85	76.10
DACB	89.05	89.93	88.69	89.28	85.24

**Table 9:** The performance of the DACB model based on 10-fold cross-validation (SEED-IV dataset).

Methods	Accuracy (%)	Precision (%)	Recall (%)	F1-Score (%)	MCC (%)
CNN-RNN	62.31	62.53	62.30	62.19	49.87
CNN-Bi-LSTM	92.87	92.89	92.87	92.87	90.50
1D CAE	85.21	85.28	85.21	85.22	80.30
1D InceptionV1	77.60	77.66	77.60	77.59	70.15
Adaboost	35.93	36.00	35.93	35.82	14.61
Bayes	25.77	28.84	25.77	17.34	1.66
XGBoost	80.57	80.81	80.57	80.61	74.15
DACB	99.73	99.72	99.72	99.72	99.64

**Table 10:** The performance of the DACB model based on 10-fold cross-validation (DREAMER-Valence).

Methods	Accuracy (%)	Precision (%)	Recall (%)	F1-Score (%)	MCC (%)
CNN-RNN	26.18	24.98	20.09	8.50	1.76
CNN-Bi-LSTM	33.09	37.75	30.25	28.77	14.60
1D CAE	50.68	52.91	49.31	49.91	37.53
1D InceptionV1	28.13	37.67	24.19	19.11	7.76
Adaboost	32.95	32.72	29.73	28.87	13.72
Bayes	21.88	21.18	21.11	13.77	2.24
XGBoost	75.90	77.08	75.15	75.89	69.48
DACB	84.26	84.39	84.03	84.18	80.12

**Table 11:** The performance of the DACB model based on 10-fold cross-validation (DREAMER-Arousal).

Methods	Accuracy (%)	Precision (%)	Recall (%)	F1-Score (%)	MCC (%)
CNN-RNN	32.11	19.21	18.56	10.67	2.17
CNN-Bi-LSTM	40.53	44.69	30.57	29.97	17.06
1D CAE	51.95	54.01	45.38	46.47	33.74
1D InceptionV1	34.45	47.99	24.24	20.67	9.38
Adaboost	38.00	35.31	29.00	29.09	13.91
Bayes	12.42	24.11	24.79	12.92	4.36
XGBoost	76.96	81.75	73.75	76.89	68.97
DACB	85.40	85.54	84.63	85.04	80.49

**Table 12:** The performance of the DACB model based on 10-fold cross-validation (DREAMER-Dominance).

Methods	Accuracy (%)	Precision (%)	Recall (%)	F1-Score (%)	MCC (%)
CNN-RNN	32.41	19.17	18.26	9.94	1.23
CNN-Bi-LSTM	37.45	40.21	26.96	24.46	12.73
1D CAE	53.16	58.68	46.76	48.76	35.76
1D InceptionV1	34.03	45.28	22.51	16.78	7.00
Adaboost	36.55	32.08	26.66	25.97	11.13
Bayes	10.44	29.42	22.62	11.36	4.30
XGBoost	77.56	81.58	76.03	78.35	69.79
DACB	85.02	85.74	84.47	85.10	79.85

**Table 13:** Ablation experiments of the DACB model based on 10-fold cross-validation (SEED-IV dataset).

DataSet	Methods	Accuracy (%)	Precision (%)	Recall (%)	F1-Score (%)	MCC (%)
SEED-IV	Block1	80.88	85.34	80.91	79.79	75.80
SEED-IV	Block2	90.68	99.68	90.68	99.68	99.58
SEED-IV	DACB	99.73	99.72	99.72	99.72	99.64

**Table 14:** Ablation experiments of the DACB model based on 10-fold cross-validation (DREAMER dataset).

DataSet	Methods	Accuracy (%)	Precision (%)	Recall (%)	F1-Score (%)	MCC (%)
DREAMER (valence)	Block1	26.08	7.21	20.00	8.27	0.11
DREAMER (valence)	Block2	32.16	5.21	20.00	8.27	0
DREAMER (valence)	DACB	84.26	84.39	84.03	84.18	80.12
DREAMER (arousal)	Block1	31.88	10.37	20.00	9.67	0.31
DREAMER (arousal)	Block2	31.87	12.11	20.00	9.66	0
DREAMER (arousal)	DACB	85.40	85.54	84.63	85.04	80.49
DREAMER (dominance)	Block1	32.38	21.80	20.01	9.81	0.84
DREAMER (dominance)	Block2	32.36	6.46	20.00	9.77	0
DREAMER (dominance)	DACB	85.02	85.74	84.47	85.10	79.85

## Data Availability

Restrictions apply to the availability of these data. Data were obtained from [BCMI laboratory, zenodo] and are available [from the authors/at https://bcmi.sjtu.edu.cn/~seed/index.html and https://zenodo.org/records/546113] with the permission of BCMI laboratory, zenodo].
